# eRNA-IDO: A One-stop Platform for Identification, Interactome Discovery, and Functional Annotation of Enhancer RNAs

**DOI:** 10.1093/gpbjnl/qzae059

**Published:** 2024-08-23

**Authors:** Yuwei Zhang, Lihai Gong, Ruofan Ding, Wenyan Chen, Hao Rong, Yanguo Li, Fawziya Shameem, Korakkandan Arshad Ali, Lei Li, Qi Liao

**Affiliations:** School of Public Health, Health Science Center, Ningbo University, Ningbo 315211, China; Biomedical Big Data Center, the First Affiliated Hospital, Zhejiang University School of Medicine, Hangzhou 310003, China; Institute of Systems and Physical Biology, Shenzhen Bay Laboratory, Shenzhen 518107, China; Institute of Systems and Physical Biology, Shenzhen Bay Laboratory, Shenzhen 518107, China; Institute of Systems and Physical Biology, Shenzhen Bay Laboratory, Shenzhen 518107, China; School of Clinical Medicine, Health Science Center, Ningbo University, Ningbo 315211, China; Institute of Drug Discovery Technology, Ningbo University, Ningbo 315211, China; School of Public Health, Health Science Center, Ningbo University, Ningbo 315211, China; School of Public Health, Health Science Center, Ningbo University, Ningbo 315211, China; Institute of Systems and Physical Biology, Shenzhen Bay Laboratory, Shenzhen 518107, China; School of Public Health, Health Science Center, Ningbo University, Ningbo 315211, China

**Keywords:** Enhancer RNA, Identification, Interactome, Functional annotation, Web server

## Abstract

Growing evidence supports the transcription of enhancer RNAs (eRNAs) and their important roles in gene regulation. However, their interactions with other biomolecules and their corresponding functionality remain poorly understood. In an attempt to facilitate mechanistic research, this study presents eRNA-IDO, the first integrative computational platform for the identification, interactome discovery, and functional annotation of human eRNAs. eRNA-IDO comprises two modules: eRNA-ID and eRNA-Anno. Functionally, eRNA-ID can identify eRNAs from *de novo* assembled transcriptomes. eRNA-ID includes eight kinds of enhancer makers, enabling users to customize enhancer regions flexibly and conveniently. In addition, eRNA-Anno provides cell-/tissue-specific functional annotation for both new and known eRNAs by analyzing the eRNA interactome from prebuilt or user-defined networks between eRNAs and protein-coding genes. The prebuilt networks include the Genotype-Tissue Expression (GTEx)-based co-expression networks in normal tissues, The Cancer Genome Atlas (TCGA)-based co-expression networks in cancer tissues, and omics-based eRNA-centric regulatory networks. eRNA-IDO can facilitate research on the biogenesis and functions of eRNAs. The eRNA-IDO server is freely available at http://bioinfo.szbl.ac.cn/eRNA_IDO/.

## Introduction

Over the past decade, a growing number of studies have reported the pervasive transcription of non-coding RNAs (ncRNAs) from active enhancer regions, termed enhancer RNAs (eRNAs). Due to the dynamic nature of enhancer activity across different tissues and lineages, eRNA transcription exhibits high specificity in biological contexts [1]. Once regarded as “transcription noise” or “byproduct” [2], eRNAs have now been shown to play crucial roles in various biological processes and diseases, such as cardiovascular development [3] and cancer [4]. Mechanistically, eRNAs can promote enhancer–promoter loops (E–P loops) and are involved in epigenetic regulation by interacting with other biomolecules, including components of cohesion or mediator [5,6], and histone acetyltransferases CBP/p300 [4,7]. Furthermore, eRNAs interact with transcription elongation factors to facilitate the pause-release of RNA polymerase II (RNAPII), thus controlling transcription elongation.

With the growing interest in eRNA functionality, several databases have been developed to characterize the transcription and potential targets of eRNAs, such as eRNAbase [8], Human enhancer RNA Atlas (HeRA) [9], the Cancer eRNA Atlas (TCeA) [10], Animal-eRNAdb [11], and eRNA in cancer (eRic) [12]. Nonetheless, these databases only provide information on annotated eRNA loci and enhancer regions but do not allow the evaluation of novel eRNAs. Additionally, several platforms exist for functional annotation of ncRNAs, but they are not well-suited for eRNAs. For example, ncRNA functional annotation server (ncFANs) v2.0 [13] requires known ncRNA identifiers as input, but most eRNAs lack a reference ID or symbol. AnnoLnc2 [14] allows the prediction of the functions of novel long non-coding RNAs (lncRNAs) based on co-expression networks but does not consider cell/tissue specificity and does not provide eRNA-specific characteristics such as histone modification, chromatin architecture, and interactive molecules. At present, a comprehensive platform for eRNA functional annotation is still lacking.

**Figure 1:**
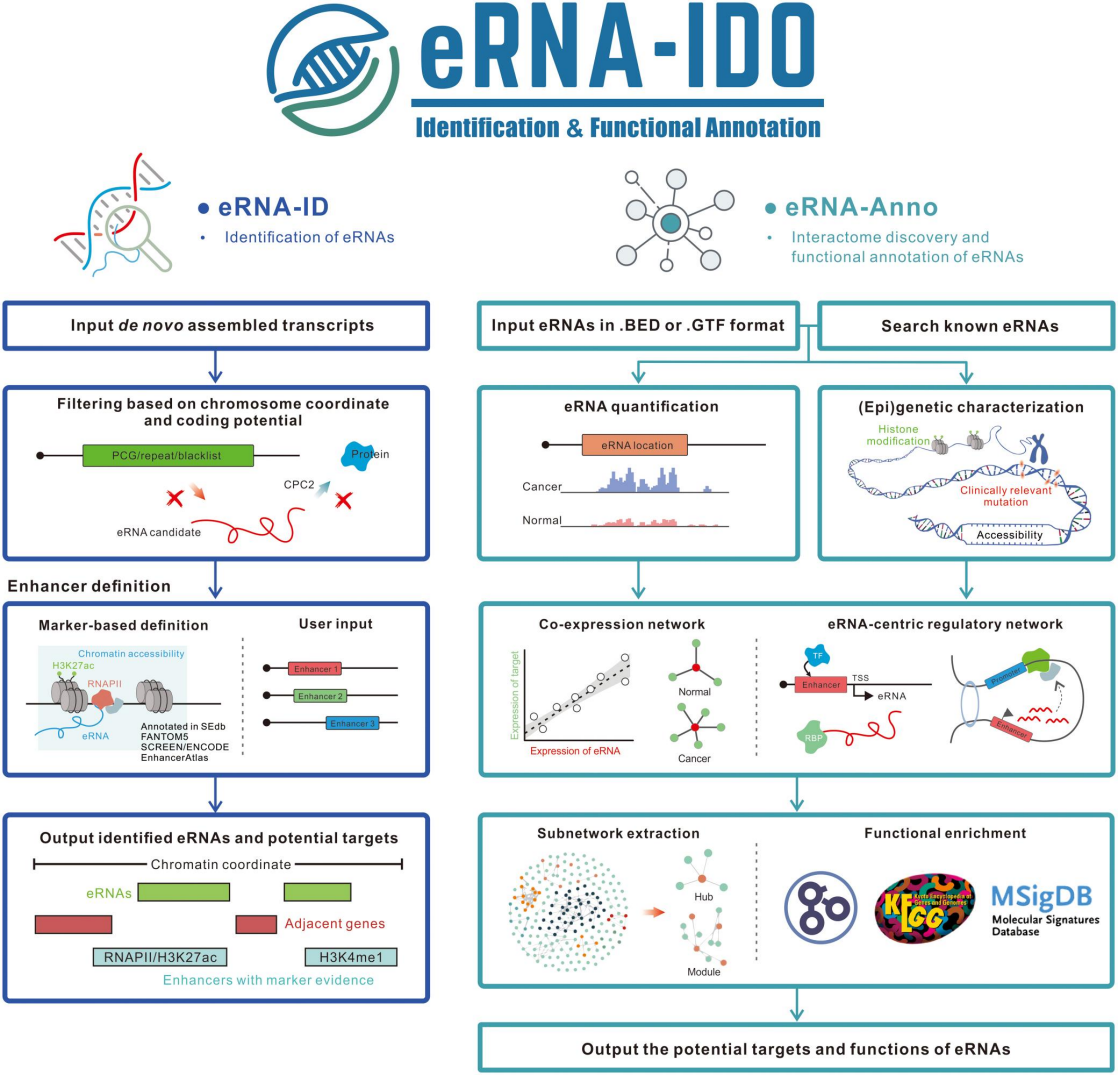
The workflow of eRNA-IDO eRNA-IDO comprises two functional modules: eRNA-ID for eRNA identification and eRNA-Anno for interactome discovery and functional annotation. eRNA, enhancer RNA; PCG, protein-coding gene; SEdb, super-enhancer database; FANTOM5, functional annotation of the mammalian genome 5; ENCODE, ENCyclopedia of DNA Elements; SCREEN, Search Candidate *cis*-Regulatory Elements by ENCODE; RNAPII, RNA polymerase II; KEGG, Kyoto Encyclopedia of Genes and Genomes; MSigDB, Molecular Signatures Database; RBP, RNA-binding protein; TSS, transcription start site; TF, transcription factor; BED, browser extensible data; GTF, gene transfer format; CPC2, Coding Potential Calculator 2.

This study introduces eRNA-IDO, the first one-stop platform for human eRNA identification, interactome discovery, and functional annotation (**Figure 1**). eRNA-IDO comprises two available modules, namely eRNA-ID and eRNA-Anno. eRNA-ID enables users to define enhancers and identify enhancer-derived ncRNAs from an uploaded *de novo* assembled transcriptome. eRNA-Anno predicts eRNA functions by discovering eRNA-connected protein-coding genes (PCGs) in normal/cancer co-expression and eRNA-centric regulatory networks. Furthermore, eRNA-IDO offers the capacity to utilize prebuilt data as well as user-defined data, providing a practical and convenient tool for biological researchers. This web server is freely available at http://bioinfo.szbl.ac.cn/eRNA_IDO/ and is open to all users without a login requirement.

## Method

### Workflow and data architecture of eRNA-ID

The schematic workflow of eRNA-ID is illustrated in Figure 1 (left panel). The processing of *de novo* assembled transcripts is initiated from user-provided RNA sequencing (RNA-seq) or global run-on sequencing (GRO-seq) data. The transcripts overlapping with annotated PCGs, simple repeats, and blacklisted regions are removed based on the GENCODE v33 reference [15]. Thereafter, the coding potential of the remaining transcripts is evaluated by Coding Potential Calculator 2 (CPC2) [16] with default parameters, and ncRNAs transcribed from enhancer regions are identified as eRNAs. Enhancer regions can be either uploaded by users in Browser Extensible Data (BED) format or defined using our marker buffet. The marker buffet comprises eight kinds of enhancer markers, including H3K27ac (Table S1), H3K4me1 (Table S2), chromatin accessibility (Table S3), RANPII binding (Table S4), super-enhancers from super-enhancer database (SEdb) 2.0 [17], EnhancerAtlas 2.0 [18] enhancers, functional annotation of the mammalian genome 5 (FANTOM5) [19] enhancers, and Search Candidate *cis*-Regulatory Elements by ENCyclopedia of DNA Elements (ENCODE) (SCREEN) [20] enhancers. The markers are optionally overlapped or merged (using BEDTools multiinter/merge) to obtain high-confidence or comprehensive enhancer profiles. The +/−3 kb regions around the center of the selected markers are defined as potential enhancer regions. These markers are cell-/tissue-specific, except those from FANTOM5 and SCREEN databases. The data type, source, and number of biosamples of these enhancer markers are listed in **Table 1**. Finally, eRNA-ID outputs the chromatin locations, adjacent genes (+/−1 Mb), and enhancers of predicted eRNAs.

**Table 1:** Data type, source, and number of biosamples of enhancer markers

Marker	Data type	Data source	No. of biosamples	Ref.
Chromatin accessibility	ATAC-seq/DNase-seq	Cistrome	371	[22]
H3K27ac	ChIP-seq	Cistrome	555	[22]
H3K4me1	ChIP-seq	Cistrome	364	[22]
RNAPII binding	ChIP-seq	Cistrome	166	[22]
FANTOM5 enhancer	–	FANTOM5	–	[19]
SCREEN enhancer	–	SCREEN	–	[20]
EnhancerAtlas enhancer	–	EnhancerAtlas 2.0	197	[18]
Super-enhancer	–	SEdb 2.0	1705	[17]

*Note*: ATAC-seq, assay for transposase-accessible chromatin using sequencing; DNase-seq, DNase I hypersensitive site sequencing; ChIP-seq, chromatin immunoprecipitation sequencing; RNAPII, RNA polymerase II; FANTOM5, functional annotation of the mammalian genome 5; ENCODE, ENCyclopedia of DNA Elements; SCREEN, Search Candidate *cis*-Regulatory Elements by ENCODE; SEdb, super-enhancer database.

### Workflow and data architecture of eRNA-Anno

The schematic workflow of eRNA-Anno is depicted in Figure 1 (right panel). The chromatin coordinates of novel eRNAs in BED/gene transfer format (GTF) format or the identifiers of known eRNAs annotated in HeRA [9] and eRic [12] databases are input in eRNA-Anno. For known eRNAs, the ENSR identifiers, chromatin coordinates, and adjacent genes (within +/−1 Mb) are accepted. Below is a detailed description of each procedure.

#### eRNA quantification

The expression levels of known eRNAs are obtained from HeRA and eRic. When chromatin coordinates of novel eRNAs are input, RNA-seq data from TCGA (https://portal.gdc.cancer.gov/) and GTEx portal [21] are used to quantify eRNA expression. Subsequently, eRNA expression levels are estimated based on the read coverage from BigWig files to shorten the processing time using the following formula:

$$\mathrm{FPKM}=\frac{\sum \left(\mathrm{Cov}\right)\times 10^{9}}{R\times L\times T}$$

where ∑(Cov) represents the total read coverage of a given eRNA region, *R* is read length, *L* is eRNA length, and *T* indicates the total mapped reads of the library.

#### Profiling genetic/epigenetic landscape

eRNA-Anno portrays a genetic/epigenetic landscape for eRNAs, including chromatin accessibility, clinically relevant mutation, and histone modification (H3K27ac and H3K4me1). Histone modification and chromatin accessibility are characterized based on chromatin immunoprecipitation sequencing (ChIP-seq) data and assay for transposase-accessible chromatin using sequencing (ATAC-seq)/DNase I hypersensitive site sequencing (DNase-seq) data, respectively, from the Cistrome Data Browser [22] (Table S1–S3). Finally, clinically relevant mutations within the query eRNA regions are collected from ClinVar [23] and the Catalogue Of Somatic Mutations In Cancer (COSMIC) [24] database.

#### eRNA–PCG network construction

Thereafter, a co-expression network between eRNAs and PCGs and an eRNA-centric regulatory network are constructed. The connected genes in the co-expression network are defined as the potential interactome of eRNAs. Both user-uploaded expression matrix and publicly available data are supported for the co-expression network. Publicly available data refer to RNA-seq data of 52 normal tissues from the GTEx portal [21] and 31 cancer types from the TCGA portal (Table S5). In addition, the toolkit GCEN [25] is used to calculate Spearman correlation coefficients and adjusted *P* values. The significant eRNA–PCG pairs are selected to construct the co-expression network according to user-defined thresholds.

For the eRNA-centric regulatory network, the relationships between eRNAs and transcription factors (TFs), RNA-binding proteins (RBPs), and E–P loops are analyzed. The eRNA–TF interactions are identified based on 11,356 ChIP-seq datasets from the Cistrome Data Browser [22], which involve 1354 TFs and 642 cells/tissues (Table S4). Furthermore, the eRNA–RBP interactions are obtained based on 518 cross-linking immunoprecipitation sequencing (CLIP-seq) datasets from the post-transcriptional regulation coordinated by RBP (POSTAR3) database [26], which involve 221 RBPs and 34 cells/tissues (Table S6). TFs and RBPs with peaks located within eRNA regions are defined as potential regulators of eRNAs. E–P loops identified by 198 HiChIP experiments across 108 cell types (Table S7) are collected from HiChIPdb [27]. The loops harboring anchors overlapping with query eRNAs are defined as eRNA-mediated loops.

#### Subnetwork extraction

Subsequently, eRNA-Anno extracts hubs/modules from the overall network to obtain tightly connected PCGs of query eRNAs. During this process, SPICi [28] in the unweighted mode (with default parameters) is utilized for module extraction.

#### Functional enrichment analyses

Functional enrichment analyses, including Gene Ontology (GO), Kyoto Encyclopedia of Genes and Genomes (KEGG) pathway, and Molecular Signatures Database (MSigDB) hallmark enrichment [29], are performed based on hypergeometric tests using our in-house scripts (https://github.com/zhangyw0713/FunctionEnrichment).

## Results

### Web interface of eRNA-ID

eRNA-ID is designed for eRNA identification based on *de novo* assembled transcriptomes. In the input interface (http://bioinfo.szbl.ac.cn/eRNA_IDO/eRNA-ID), users are required to upload a transcriptome profile in GTF format, which can be generated from RNA-seq and GRO-seq data, and define enhancer regions using either provided marker buffet or by uploading a customized BED file. eRNA-ID adopts an analytical workflow similar to ncFANs-eLnc [13] to identify eRNAs (see Method). As shown in Table S8, the major advantage of eRNA-ID over ncFANs is its prebuilt buffet of eight kinds of enhancer markers (H3K27ac, H3K4me1, chromatin accessibility, RNAPII binding, SEdb 2.0 super-enhancers [17], and three types of enhancer annotations from EnhancerAtlas 2.0 [18], FANTOM5 [19], and SCREEN [20] databases), enabling users to customize enhancer regions of interest. For example, users may require high-confidence enhancer regions simultaneously labeled by multiple markers or may want to obtain as many enhancers as possible by merging multiple markers. The processing procedure of eRNA-ID is fast. For instance, a GRO-seq-derived transcriptome with 3483 transcripts (SRA008244) took 45 s, and a RNA-seq-derived *de novo* transcriptome with 222,848 transcripts (GSM2824220) took 88 s (default parameters).

**Figure 2:**
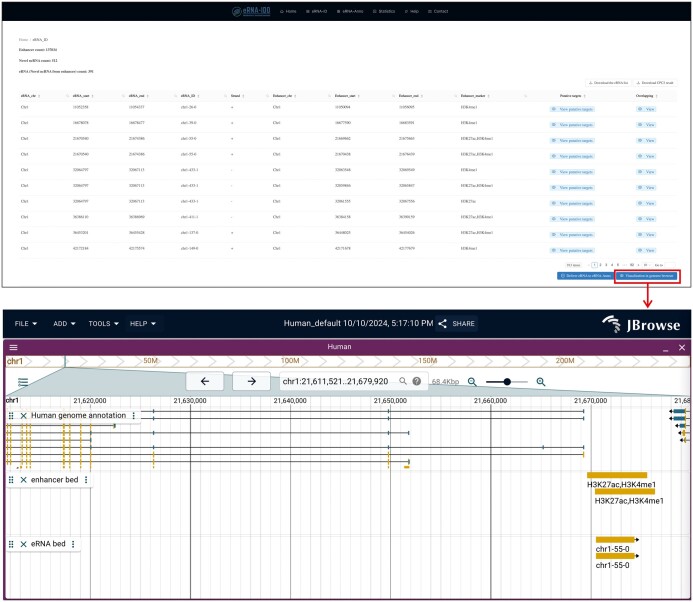
The output interface of eRNA-ID The predicted eRNA locations, enhancer regions, markers for active enhancers, putative targets (adjacent genes), and overlapping lncRNAs are displayed in a table and can be visualized in the genome browser. Additional details are shown in the demo: http://bioinfo.szbl.ac.cn/eRNA_IDO/retrieve/?taskid=5a9LFXS8oGCm. lncRNA, long non-coding RNA.

In the output interface of eRNA-ID (**Figure 2**), the chromatin coordinates, enhancers, and putative targets (adjacent genes within +/−1 Mb of eRNAs) of identified eRNAs are displayed in a table. Users can also view the information in a genome browser based on JBrowse [30]. Moreover, functional annotation can be conducted for these novel eRNAs by clicking on the “Deliver eRNA to eRNA-Anno” button.

### Web interface of eRNA-Anno

**Figure 3:**
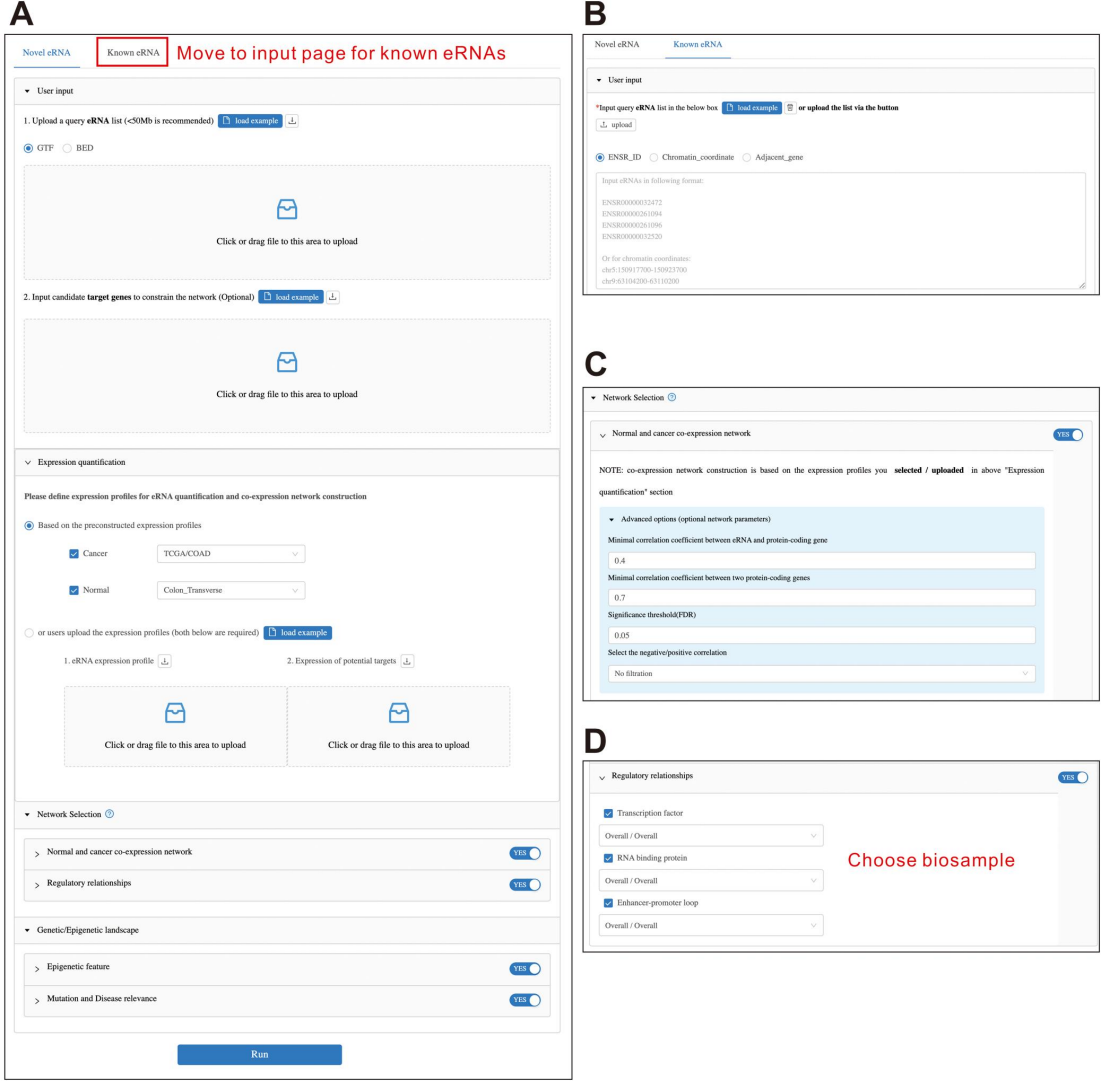
The input interface of eRNA-Anno **A**. The input contents include a potential eRNA list, optional target candidates, parameters for eRNA quantification, network selection, and genetic/epigenetic landscape. **B**. The input interface for known eRNAs annotated in HeRA [9] and eRic [12]. **C**. Parameters for the construction of the co-expression network. **D**. Parameters for the construction of the eRNA-centric regulatory network. HeRA, Human enhancer RNA Atlas; eRic, eRNA in cancer.

eRNA-Anno is designed for the network-based interactome discovery and functional annotation of eRNAs. In this module, users input the chromatin coordinates of novel eRNAs (**Figure 3**A) or the identifiers/locations of known eRNAs annotated in HeRA [9] and eRic [12] databases (Figure 3B), followed by network selection and parameter setting. eRNA-Anno first quantifies the eRNA expression levels based on RNA-seq data from TCGA and GTEx portal. As hundreds of RNA-seq samples require a long processing time, the read coverages from BigWig files were used to speed up the quantification (see Method). To examine the reliability of this method, the expression levels of known eRNAs acquired via this method were correlated with those based on the canonical featureCounts [31] method obtained from HeRA and eRic databases. The results reveal that our method is highly correlated with the canonical method (Figure S1A and B) and is approximately 400 times faster (Figure S1C).

eRNA-Anno annotates the functions of eRNAs by discovering their interactomes based on eRNA–PCG networks, including normal co-expression networks based on GTEx expression profiles [21], cancer co-expression networks based on TCGA expression profiles (https://portal.gdc.cancer.gov/), and eRNA-centric regulatory networks. Co-expression relationships are widely used to annotate the functions of eRNAs [32–34]. Additionally, eRNAs have been reported to exert regulatory functions by interacting with other biomolecules, such as TFs [35–37], RBPs [4,38,39], and target genes activated by E–P loops [40,41]. Therefore, the regulatory network can be used for eRNA functional annotation, similar to approaches used for other ncRNAs [13,42–45]. The network construction procedure is detailed in the Method section. Parameters include tissue/cancer type of expression profile, co-expression coefficient, significance threshold, biosamples of interaction relationships, and epigenetic landscape (Figure 3C and D).

Upon receiving launch instructions, eRNA-Anno initiates the analytical procedure (see Method) to identify the potential targets of query eRNAs from selected networks and annotate their functions based on hub-based and module-based strategies. The whole procedure typically takes tens of minutes, depending on the number of input eRNAs (Figure S2). Hence, users are recommended to set an email notification or record the task ID for result retrieval when submitting a task with a large set of eRNAs.

**Figure 4:**
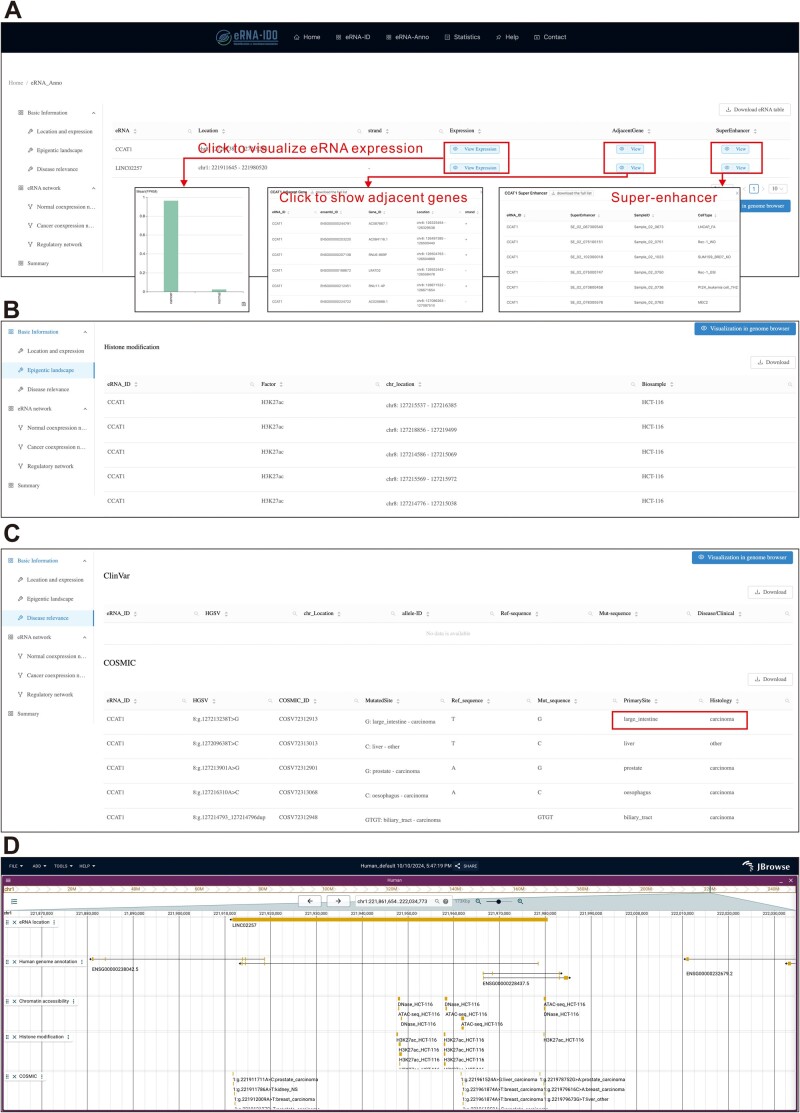
The output interface of eRNA-Anno showing the basic information of query eRNAs CCAT1 and LINC02257 **A**. The locations and expression levels of CCAT1 and LINC02257. **B**. The epigenetic landscape. **C**. Clinically relevant mutations within the genomic regions of CCAT1 and LINC02257. **D**. The genome browser visualization accessible via the “Visualization in genome browser” button. Further details are shown in the demo: http://bioinfo.szbl.ac.cn/eRNA_IDO/retrieve/?taskid=97XPLicEAj4euYG/.

**Figure 5:**
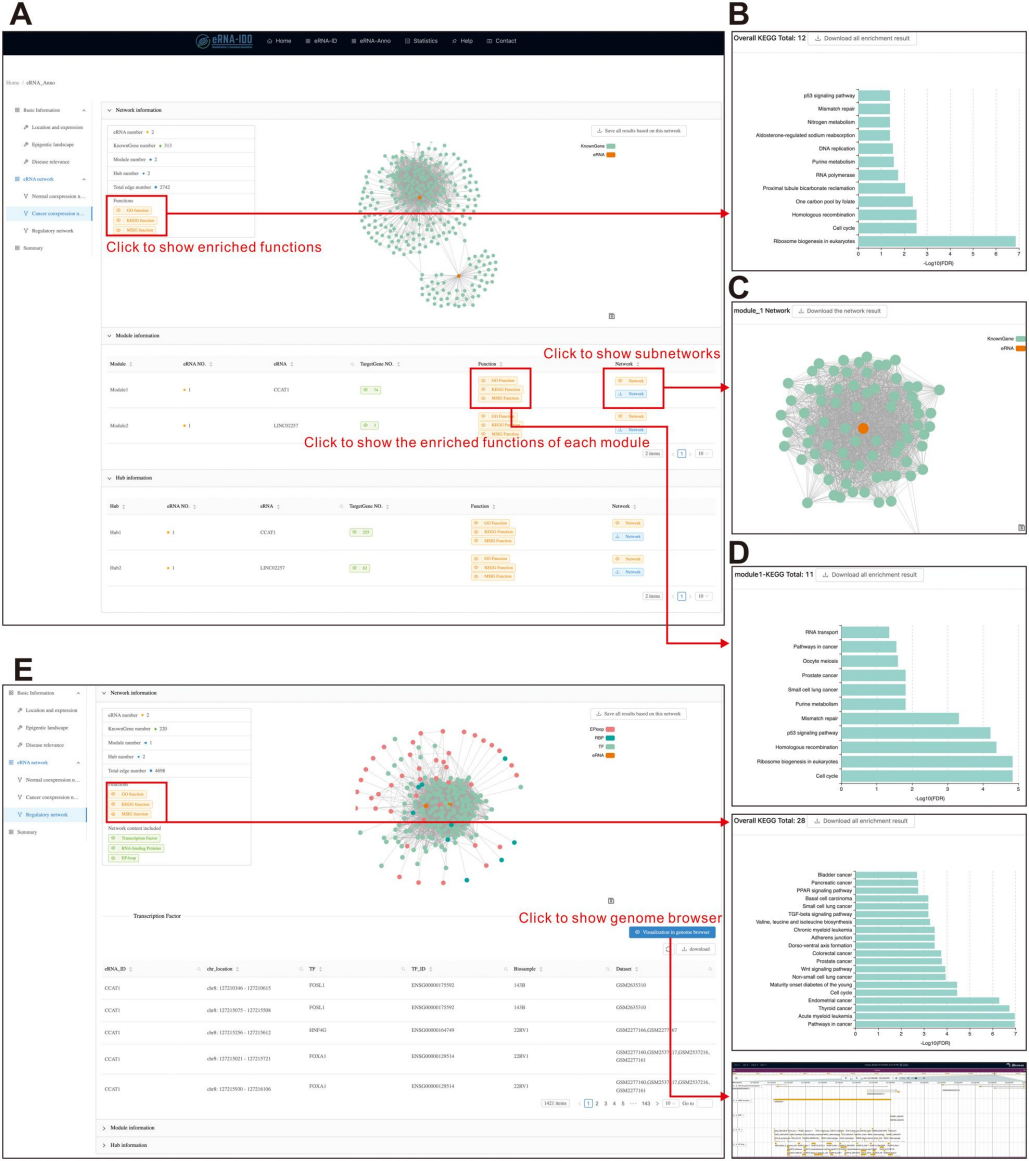
The output interface of eRNA-Anno showing the interactomes and functions of CCAT1 and LINC02257 **A**. The co-expression network of CCAT1 and LINC02257 in human colorectal cancer. **B**. The enriched KEGG pathways of CCAT1- and LINC02257-connected PCGs. **C**. Visualization of the CCAT1-containing module. **D**. The enriched KEGG pathways of PCGs within the CCAT1-containing module. **E**. The CCAT1-centric and LINC02257-centric regulatory network.

In the output interface, eRNA-Anno provides basic information about eRNAs (*i.e.*, location and expression, epigenetic landscape, and disease relevance) and putative targets and functions based on various networks. In the “Location and expression” section, chromatin coordinates, expression levels in normal and cancer samples, adjacent genes (≤ 1 Mb), and overlapping super-enhancers are listed in the table (**Figure 4**A). Furthermore, eRNA-Anno profiles active enhancer markers (H3K27ac and H3K4me1) and chromatin accessibility of eRNA regions to evaluate the activity of enhancers where eRNAs are transcribed (Figure 4B). Considering that mutations in eRNA regions are often related to eRNA expression and subsequent disease development [46], clinically relevant mutations within query eRNA regions are displayed in the “Disease relevance” section (Figure 4C) and can be visualized in the genome browser (Figure 4D). Moreover, the interactome and predicted functions of eRNAs based on the selected networks are displayed in the second part (**Figure 5**). For example, in a cancer co-expression network (Figure 5A), the eRNA–PCG network is visualized in a force-directed layout, and the functions of connected PCGs are provided (Figure 5B). Since genes with similar functions tend to be concentrically distributed, eRNA-Anno extracts hubs and modules composed of tightly connected genes from the overall network (Figure 5C). The functions of query eRNAs can be inferred by the functions of the PCGs within the same module or hub (Figure 5D).

**Figure 6:**
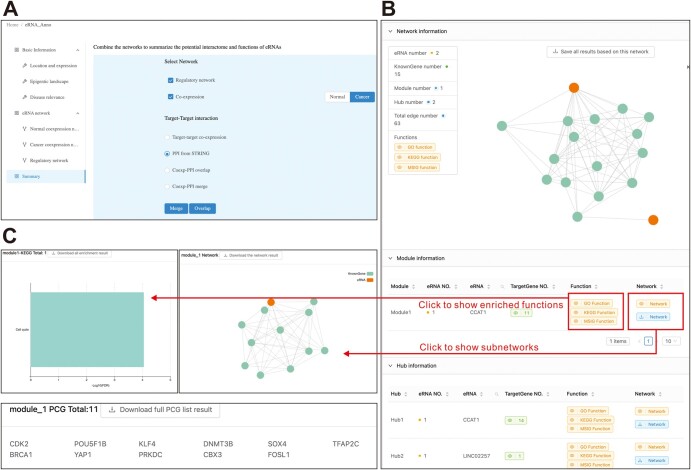
Summary of the interactome and functions of query eRNAs based on the combination of co-expression network and regulatory network **A**. Parameter settings for network combination. **B**. A high-confidence network comprising the overlapping nodes and edges generated for CCAT1 and LINC02257. **C**. The CCAT1-containing module indicating its interactive genes and functions in cell cycle regulation.

In addition, the eRNA-centric regulatory network (Figure 5E) provides a visualization of the relationships of eRNAs with TFs, RBPs, and E–P loops in multiple modes, including network topology, table, and genome browser. Similarly, the functions of eRNAs can be inferred by the related biomolecules in the overall network, modules, or hubs. The results of individual networks can be combined into a summary (**Figure 6**).

### A case study demonstrating the usage of eRNA-Anno

Since the input interface has many user-dependent options and the output interface displays interactive information, a case study is described to introduce the usage and interpret the results obtained from eRNA-Anno. CCAT1 and LINC02257, which have been characterized as colon cancer-associated eRNAs [47,48], were analyzed in this study and input in GTF format. Subsequently, “TCGA-COAD” and “GTEx-Colon Transverse” were chosen, co-expression and regulatory networks were selected, the parameters were set, and eRNA-IDO was launched, as depicted in Figure 3.

In the output interface, eRNA-Anno revealed that both CCAT1 and LINC02257 exhibited higher expression levels in colorectal cancer (Figure 4A) and showed enriched active enhancer markers (Figure 4B), which is consistent with previous studies [47,48]. Additionally, the genomic regions of CCAT1 and LINC02257 harbor carcinoma-associated mutations (Figure 4C), indicating their clinical significance. Subsequently, the co-expression network in colon adenocarcinoma was further investigated to evaluate the interactome and functions of CCAT1 and LINC02257. The topology of the co-expression network revealed limited connections between CCAT1 and LINC02257 (Figure 5A), indicating their independent regulatory roles. Furthermore, functional enrichment analysis of the co-expressed PCGs revealed that CCAT1 and LINC02257 were potentially enriched in translation and cell cycle pathways (Figure 5B). The CCAT1-containing module precisely pinpointed the role of CCAT1 in regulating cell cycle (Figure 5C and D), which conforms to previous findings [30,49]. Moreover, the eRNA-centric regulatory network detected the interactive TFs, RBPs, and genes involved in E–P loops. These interactive molecules were enriched in cell cycle and cancer pathways, suggesting the similar role of CCAT1 and LINC02257 (Figure 5E). Additionally, a genome browser based on JBrowse [30] visualized eRNA locations and the mutational, epigenetic, and interactive landscapes (Figure 5E). Finally, the nodes and edges from two separate networks were overlapped to determine high-confidence interactions of CCAT1 in a cell cycle-related module (Figure 6), including some previously reported targets such as CDK4 [50] and SOX4 [51]. This case study demonstrates the application of eRNA-Anno, displaying its ability to comprehensively and reliably predict eRNA interactome and functions.

## Discussion

As a web server dedicated to eRNA analysis, eRNA-IDO provides a convenient method for eRNA identification, interactome discovery, and functional annotation. The major advantages of eRNA-IDO include but are not limited to the following. First, eRNA-ID includes eight kinds of enhancer markers, offering a more convenient and customized approach for enhancer definition compared to ncFANs-eLnc [13], which only includes the H3K27ac marker. Second, eRNA-Anno is applicable to both novel and known eRNAs. Considering the poor characterization of eRNAs, the applicability to novel eRNAs grants eRNA-Anno higher flexibility and biological practicability compared to other tools requiring known identifiers (such as ncFANs v2.0 [13]) and other databases [9–12]. The detailed comparison between eRNA-IDO and ncFANs v2.0 is displayed in Table S8. Third, biological context-specific expression and interaction profiles are prebuilt in eRNA-Anno. Compared to tools without biological specificity such as AnnoLnc2 [14], eRNA-Anno is expected to provide more precise findings for *in vivo* investigations. Moreover, the prebuilt profiles facilitate the use of the service. Finally, eRNA-IDO is the first one-stop platform for eRNA identification, interactome discovery, and functional annotation.

Nevertheless, the limitations of the study should be acknowledged and overcome. First, eRNA-IDO is currently designed for human data, and additional species will be supported in the future. Second, some characteristics such as the m^6^A modification [52] and RNA structure [53,54] are essential for eRNA functionality but are not evaluated by eRNA-IDO. Third, the current iteration of eRNA-IDO only considers normal tissue and cancer. In the future, a larger number of disease-specific and cell-specific expression and interaction profiles will be incorporated. Hopefully, eRNA-IDO will benefit from user feedback and develop into a more powerful tool upon continuous updates.

## Supplementary Material

qzae059_Supplementary_Data

## Data Availability

The eRNA-IDO web server is available at http://bioinfo.szbl.ac.cn/eRNA_IDO/.

## CRediT author statement

**Yuwei Zhang:** Data curation, Methodology, Investigation, Writing – original draft. **Lihai Gong:** Methodology, Software, Visualization. **Ruofan Ding:** Data curation, Visualization. **Wenyan Chen:** Data curation, Investigation. **Hao Rong:** Data curation, Investigation. **Yanguo Li:** Data curation. **Fawziya Shameem:** Writing – review & editing. **Korakkandan Arshad Ali:** Writing – review & editing. **Lei Li:** Resources, Conceptualization, Supervision, Writing – review & editing. **Qi Liao:** Conceptualization, Supervision, Writing – review & editing, Funding acquisition. All authors have read and approved the final manuscript.
