# Supplementary material for: eRNA-IDO: A One-stop Platform for Identification, Interactome Discovery, and Functional Annotation of Enhancer RNAs
Source: Genomics Proteomics Bioinformatics. 2024 Aug 23;22(4):qzae059. doi: 10.1093/gpbjnl/qzae059 (PMC11514848; doi:10.1093/gpbjnl/qzae059)
Supplement: qzae059_Supplementary_Data [file qzae059_supplementary_data.zip › supplementary material captions.docx]

**Supplementary material**

**Figure S1 Comparison of the strategies for eRNA quantification**

**A.** Distribution of Pearson correlation coefficients of eRNA levels quantified by eRNA-IDO (see Method) with those collected from the HeRA database. **B.** Distribution of Pearson correlation coefficients of eRNA levels quantified by eRNA-IDO with those collected from the eRic database. The labels on y-axis are short for cancer types in TCGA database (https://gdc.cancer.gov/resources-tcga-users/tcga-code-tables/tcga-study-abbreviations). **C.** Comparison of the processing time between our method and the canonical featureCounts method [31]. The test sample is GTEX-ZYFC-2626-SM-5NQ6S from GTEx database. The task was performed on a Dell Precision T7920 workstation with a single core. GTEx, Genotype-Tissue Expression; TCGA, The Cancer Genome Atlas.

**Figure S2 The processing time of eRNA-Anno based on different eRNA numbers**

The X-axis and Y-axis represent the input eRNA numbers and the running time, respectively.

**Table S1 ChIP-seq datasets of H3K27ac modification**

**Table S2 ChIP-seq datasets of H3K4me1 modification**

**Table S3 ATAC-seq/DNase-seq datasets of chromatin accessibility**

**Table S4 ChIP-seq datasets of 1354 transcription factors**

**Table S5 Normal tissue-specific and cancer-specific RNA sequencing (RNA-seq) datasets.**

**Table S6** **CLIP-seq datasets of RNA binding proteins**

**Table S7** **HiChIP datasets from HiChIPdb**

**Table S8 Comparison between eRNA-IDO and ncFANs v2.0**
