# Supplementary material for: eRNA-IDO: A One-stop Platform for Identification, Interactome Discovery, and Functional Annotation of Enhancer RNAs
Source: Genomics Proteomics Bioinformatics. 2024 Aug 23;22(4):qzae059. doi: 10.1093/gpbjnl/qzae059 (PMC11514848; doi:10.1093/gpbjnl/qzae059)
Supplement: qzae059_Supplementary_Data [file qzae059_supplementary_data.zip › TableS8-done.docx]

**Table S8 Comparison between eRNA-IDO and ncFANs v2.0**

|  | eRNA-IDO | ncFANs v2.0 |
| --- | --- | --- |
| Pros | 1. eRNA-ID includes 8 kinds of markers for flexible enhancer definition. 2. eRNA-Anno provides functional annotations for both new and known eRNAs 3. eRNA-Anno provides eRNA-specific characteristics such as histone modification and chromatin architecture. | 1. ncFANs-eLnc can be used for both human and mouse eRNA identification. |
| Cons | 1. eRNA-ID can only be used for human eRNA identification. | 1. ncFANs-eLnc includes only H3K27ac marker for enhancer definition. 2. ncFANs-NET provides functional annotations for only annotated ncRNAs with known identifiers. 3. ncFANs cannot provide any eRNA-specific characteristics. |

*Note*: eRNA, enhancer RNA; ncFANs, ncRNA functional annotation server.
