# Supplementary figures and images for: eRNA-IDO: A One-stop Platform for Identification, Interactome Discovery, and Functional Annotation of Enhancer RNAs
Source: Genomics Proteomics Bioinformatics. 2024 Aug 23;22(4):qzae059. doi: 10.1093/gpbjnl/qzae059 (PMC11514848; doi:10.1093/gpbjnl/qzae059)

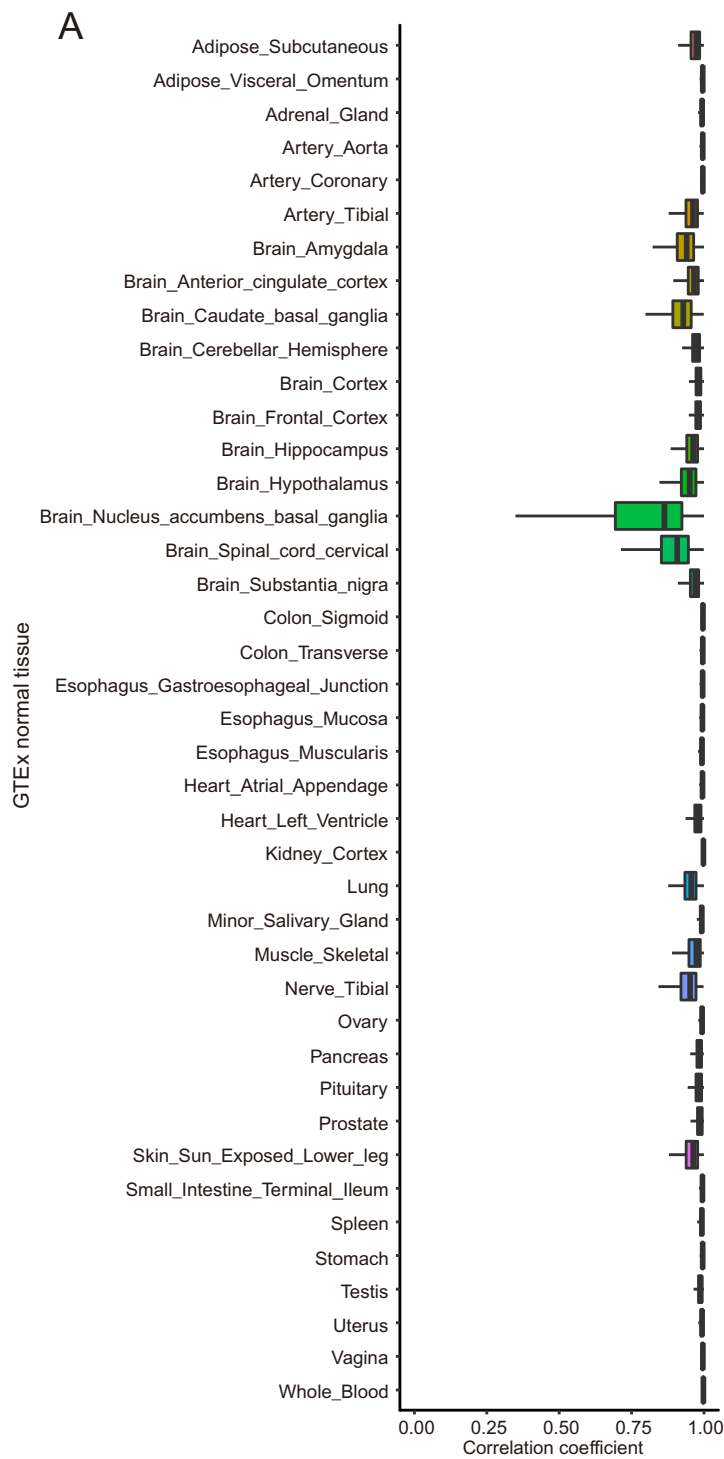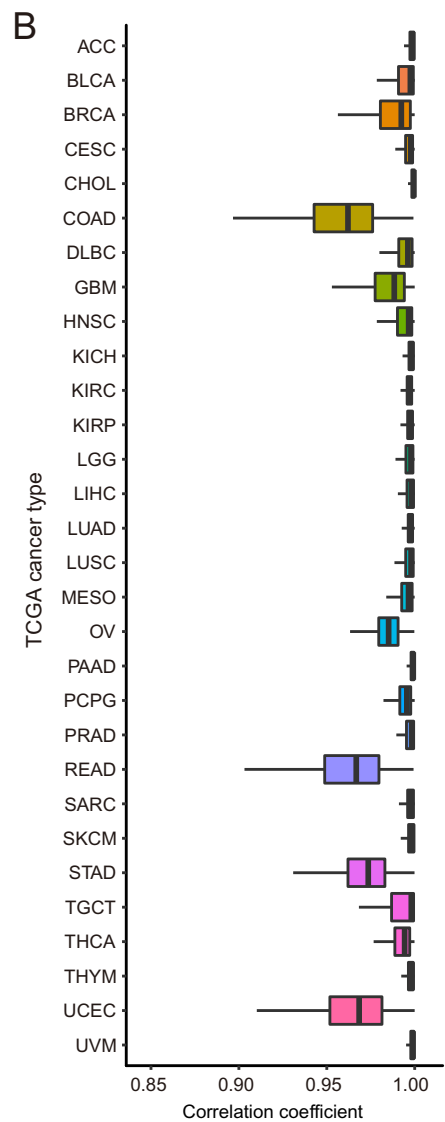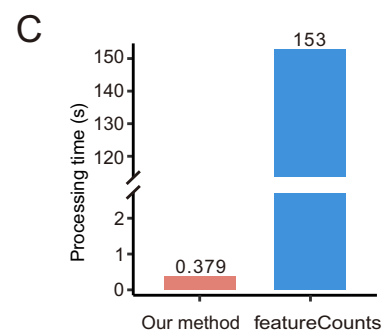

Supplement: qzae059_Supplementary_Data [file qzae059_supplementary_data.zip › FigS1.pdf]

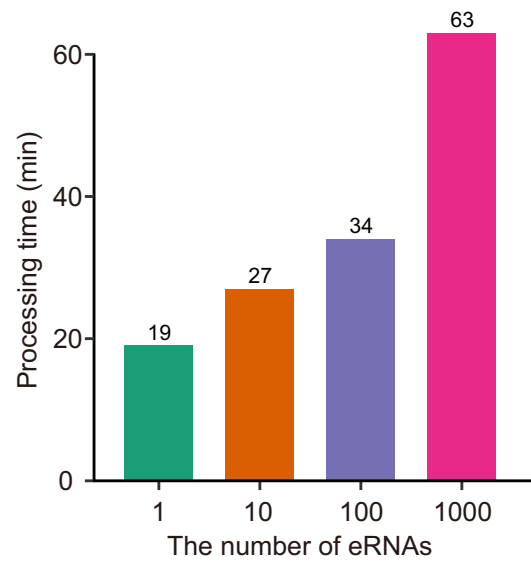

Supplement: qzae059_Supplementary_Data [file qzae059_supplementary_data.zip › FigS2.pdf]
